# Colorectal cancers associated with mismatch repair deficiency

**DOI:** 10.3389/fmed.2025.1649565

**Published:** 2025-09-08

**Authors:** Mingzhu Sun, Kevin Monahan, Jayne Moquet, Stephen Barnard

**Affiliations:** ^1^Cytogenetics Group, Radiation Effects Department, Radiation, Chemical, Climate and Environmental Hazards Directorate (RCCE), UK Health Security Agency (UKHSA), Didcot, United Kingdom; ^2^Lynch Syndrome Clinic, Centre for Familial Intestinal Cancer, St Mark's Hospital, London North West University Healthcare NHS Trust, Harrow, United Kingdom; ^3^Department of Surgery and Cancer, Imperial College London, South Kensington Campus, London, United Kingdom

**Keywords:** colorectal cancer, mismatch repair deficiency, lynch and lynch-like syndrome, microsatellite instability, immune checkpoint inhibitor, radiation effects

## Abstract

This review aims to summarize the latest updates in the prevention, diagnosis, treatment and management of colorectal cancers (CRCs) associated with mutation(s) or alteration(s) in the DNA mismatch repair (MMR) genes. It covers inheritable (Lynch syndrome, constitutional MMR-deficiency syndrome and Familial CRC X) and sporadic CRCs as well as Lynch-like syndrome with both heritable and sporadic features. Despite recommendation for universal testing in all newly diagnosed CRCs, cases with MMR deficiency (dMMR) are still generally underdiagnosed and undertreated in current clinical practice. The distinct molecular and clinicopathologic features of this unique subset of CRC have significant medical importance and have attracted continuous research interest over the years. Updated and currently ongoing research have shown promising results associated with improved clinical outcomes for these patients.

## 1 Mismatch repair (MMR)

As cells replicate in the mucosa, genetic mutations accumulate in the colon and rectum tissues over time leading to tumorigenesis. Most tumors contain 100–200 mutations; however, ~15% of CRC tumors may contain thousands of mutations and are described as “hypermutated” tumors. These tumors typically harbor genetic or epigenetic alterations in the MMR genes causing rapid accumulation of mutations (1).

Evolutionarily, the MMR system is a highly conserved DNA repair system that functions in the maintenance of genomic integrity primarily by removing base-base mismatches and erroneous insertion/deletion (indel) loops generated during DNA replication or recombination (2–4). It corrects replication errors in newly synthesized DNA and prevents recombination between non-homologous sequences. Overall, the MMR system can increase replication fidelity by ~100- to 1,000-fold (5, 6). The MMR system involves many proteins (~20) and can repair DNA tracts up to 2 kb (3); and it repairs DNA in a strand-specific and bi-directional manner (4).

The mismatch repair mechanism has been broadly established within literature with illustrated pathways (4, 7, 8). In brief, MutSα (MSH2 and MSH6) or MutSβ (MSH2 and MSH3) heterodimers that form in response to DNA damage recognize and bind to the mismatched bases or indel loops of preferred sizes; namely, MSH2/MSH6 for single base mismatch and small indel loops and MSH2/MSH3 for larger size extrusions. In the presence of ATP, the MutS heterodimer undergoes a conformational change, which in turn recruits the second heterodimer MutLα that is comprised of MutL homolog 1 (MLH1) and postmeiotic segregation increased 2 (PMS2). Thus, a dynamic ATP-dependent DNA-MutSα/β-MutLα ternary complex is assembled that regulates the downstream steps of repair. This complex, together with many other functional proteins (such as proliferating cell nuclear antigen, replication factor C, exonuclease 1, replication protein A, DNA polymerase δ, and DNA ligase I) (4), diffuse along the DNA and connect to a strand scission site and subsequently stimulate a latent endonuclease activity of PMS2. PMS2 introduces single-strand nicks into the daughter DNA and the mismatch is removed by exonuclease 1 excision. The resulting single-strand gap is then filled by a replicative polymerase holoenzyme complex and the nick is finally sealed by DNA ligase (9, 10). Inactivation of *MSH3* has not yet been conclusively linked to an elevated cancer risk in humans, and therefore, this gene will not be further discussed in this review.

MMR proteins have been reported to participate directly or indirectly in recombination suppression, DNA damage recognition and signaling, G2/M phase cell cycle arrest, nucleotide excision, repair of clustered DNA damage, cytotoxicity, and apoptosis (11, 12) as well as immunoglobulin gene rearrangement (4). In addition, MMR is involved in class-switch recombination, interstrand DNA cross-link repair and the repair of aberrant triple-repeat expansions (13). Furthermore, MMR is also linked to the generation of mutations associated with Turcot syndrome (1), Huntington's disease and other neurodegenerative disorders (10). The mechanisms implicated in these processes remain largely unknown.

## 2 Different types of CRC with dMMR

### 2.1 Inheritable CRC with dMMR

#### 2.1.1 Lynch syndrome (LS)

LS is an autosomal dominant hereditary condition that accounts for ~3–5% of all cases of CRC and is associated with an increased accumulative lifetime risk of CRC development depending on the variant of MMR mutation and age (14). LS is a pan-cancer predisposition syndrome associated with both synchronous and metachronous cancers (15). In LS patients, primary tumors can also arise at various extracolonic organs with a higher rate of cellular turnover, such as the endometrium, stomach, small bowel, hepatobiliary system, upper urologic tract and ovary (9) albeit less frequently than CRC. The median age for the onset of cancer in LS patients is 45 years, which is approximately two decades earlier than in sporadic CRC (16). Typically, LS-associated CRC has accelerated carcinogenesis (2–3 years) in comparison to sporadic CRC (6–10 years) (9).

LS is characterized by heterozygous pathogenic mutation(s) in one or more (very rarely) of the DNA MMR genes with clinical relevance to CRC, chiefly *MLH1, MSH2, MSH6* and *PMS2*, or large deletion in the 3′ terminus of the *EPCAM* (epithelial cell adhesion molecule) gene that silences *MSH2* expression (17). MMR deficiency (dMMR) may occur through the loss of heterozygosity, epigenetic modification (such as methylation), or point mutation (18). Somatic inactivation of the remaining wild-type allele of these genes are routinely found in LS-associated tumors (19). Germline mutation in different MMR genes is correlated with various phenotypic traits. For example, cases with mutations in *MLH1* or *MSH2* typically develop LS phenotypes with early onset age and family history. Cases with *MSH2* mutations tend to develop more extracolonic tumors and also predominate in the Muir-Torre variant of LS. In contrast, germline mutations in *MSH6* or *PMS2* tend to develop an atypical LS phenotype (9). Additionally, even in families with the same germline mutation, phenotypes vary between family members in terms of cancer onset age and histological and pathological features (20, 21). Not all alterations in MMR genes are pathogenic, benign polymorphisms and variants of uncertain significance have been recorded in large databases (4).

Another characteristic feature of LS is genome-wide microsatellite instability (MSI) due to the loss of activity of MMR proteins. Microsatellites are tandem repeated sequences of DNA (1 to 6 base pairs in length) (22) present in both coding and noncoding regions (4). The majority of dMMR cells develop a “mutator phenotype” with 10^2^-10^3^ fold increase in the spontaneous mutation rate that can affect the entire genome including a high frequency of frameshift mutations in microsatellite DNA repeats as the result of polymerase misincorporation errors (23, 24).

In line with the occurrence frequency, MSI can be stratified as MSI-high (MSI-H), MSI-low (MSI-L) and microsatellite stable (MSS). At present, MSI-L and MSS tend to be considered as the same category in comparison to MSI-H (25). MSI-H is typically associated with mutations in *MLH1* or *MSH2* (26, 27) and the appearance of MSI-L is largely due to mutations in *MSH6* (10%) or *PMS2* (5%) (28). Approximately 5% of MSI tumors have no identified etiological cause (29). Over 95% of LS tumors have MSI, whereas MSI only exists in 10–15% of the sporadic CRC (23). It is worth mentioning that MSI incidence is higher in early stage CRC (~20% in stages I and II and 12% in stage III) than in metastatic stage (4–5%) (30), which could be informative for treatment planning and prognosis interpretation. The prevalence of dMMR/MSI-H CRC also varies in different ethnic populations with higher rates reported in Europeans (5–24%), Caucasian Americans of European descent (8–20%), African Americans (12–45%) and Egyptians (37%); whilst relatively lower rates reported in Asian countries with 4.5–15.0% in China and 3.8–20.0% in Japan (18).

#### 2.1.2 Constitutional MMR-deficiency syndrome (CMMR-D)

In addition to the heterozygous MMR gene mutation observed in LS, biallelic deleterious germline mutations can occur in MMR genes leading to constitutional mismatch repair-deficiency (CMMR-D). CMMR-D cases are extremely rare (1 in 1,000,000 new-borns; https://genturis.eu) and usually observed in children or young adults. It is characterized by a phenotype that resembles neurofibromatosis type 1 (NF1) and a broad spectrum of high penetrance and early-onset malignancies, such as hematologic malignancy, brain tumor, LS-associated tumors and a few other types (31, 32). Biallelic germline mutations may appear in any of the four genes commonly associated with LS even though clinical features in patients with biallelic germline mutations in *MLH1* or *MSH2* differ from those with biallelic germline mutations in *MSH6* or *PMS2* (33). *PMS2* is affected in over 50% of cases, whereas *MSH2* and *MLH1* biallelic pathogenic variants account for a small proportion of cases (https://genturis.eu). Tumors with biallelic MMR variants are believed to show the same molecular phenotype as monoallelic LS tumors (34); namely, mismatch repair deficiency, mutator phenotype and microsatellite instability.

#### 2.1.3 Familial CRC X

Familial CRC X syndrome refers to patients who meet the clinical criteria for LS, but have MSS tumors (1). These patients tend to have distal cancer that appears at a later age (mid-50 years) in comparison to LS, with a reduced risk for extracolonic malignancies and an associated high degree of chromosomal instability more similar to sporadic cancers (1). Familial CRC X is less likely to be associated with synchronous or metachronous cancers; however, the 10-year risk of cancer-related death is significantly higher in these patients than in LS (15.4% vs. 8.9% in men and 19.3% vs. 8.7 in women (35).

### 2.2 Lynch-like syndrome (LLS)

Thousands of germline MMR gene variants have been identified and recorded in the InSiGHT database (36). Despite advancements in genetic screening techniques, pathogenic germline mutations in the MMR genes remain undetected for up to 30% of families with a clinical suspicion of LS (9), and unidentified genes might account for 1/3 to 1/2 of the missing heritability of CRC (37).

Cases with the presence of MSI and impaired MMR pathways in the absence of an inactivating pathogenic germline mutation in MMR genes or *MLH1* hypermethylation are defined as Lynch-like syndrome (LLS). Patients with LLS have a risk of CRC in first-degree relatives between the risk of LS and sporadic cases (38). There are four hypotheses that may explain the observed MSI and dMMR phenotype in LLS: 1) atypical germline alterations in MMR genes; 2) germline alterations in other genes associated with mismatch repair; 3) somatic alterations in other genes triggering a cascade that affects MMR expression; 4) biallelic somatic mutations in the MMR genes (39). LLS is distinguishable from sporadic CRC by the absence of epigenetic silencing of *MLH1* or mutations in the B-Raf proto-oncogene (*BRAF*) (18). LLS may be comprised of both hereditary and sporadic cases. At present no effective testing protocols nor adequate classification criteria are available for these patients (39).

### 2.3 Sporadic CRC with dMMR

In comparison to LS, sporadic CRC tumors appear at older age and develop at a slower pace (1). Typically, there is a lack of family history for sporadic CRC and in humans non-functional MMR caused by somatic promotor hypermethylation of the *MLH1* gene and its subsequently reduced expression are associated with sporadic CRC (40–42). These tumors are often accompanied with mutations in *BRAF*, specifically the V600E (43) and exhibit CpG island methylator phenotype leading to methylation of numerous gene promoters (18).

In contrast to germline loss, somatic loss of MMR, either through mutation or downregulation of gene expression, is often partial in terms of functional impact. Somatic mutations are often subclonal and overwhelmingly missense variants with unknown significance. Additionally, partial loss of MMR gene may not induce high tumor mutation burden and MSI (44) and therefore may contribute to intra-tumor heterogeneity and complicate diagnosis and treatment.

Recently, it has been reported that constitutional monoallelic methylation of the *MLH1* promoter can cause the silencing of that allele by a mechanism of transcriptional downregulation, which is cancer-predisposing and should be considered as a cause of LS and managed in the same ways as LS with sequence variants in *MLH1* (45).

Table 1 shows the mean age of onset, prevalence, cancer risk, genetic features, MSI status, and family history of different types of MMR deficient CRC.

**Table 1:** Summary for the different types of colorectal cancer (CRC) with mismatch repair (MMR) deficiency.

**Types of CRC**	**Mean age of onset (years)**	**Prevalance**	**Cancer risk**	**Genetic features**	**MSI status**	**Presence of family history**
**Lynch syndrome** (Dowty et al., 2013; Heinen, 2016; Martínez-Roca et al., 2022; Cazzaniga et al., 2025)	Mid-40	3–5% of all CRC cases	34–47% among male and 36–37% among female by age 70 years for *MLH1* and *MSH2* mutation carriers, respectively; lifetime CRC risk 50–80%	Heterozygous germline mutations in DNA MMR genes (chiefly, MLH1, MSH2, MSH6, and PMS2); germline deletions of the *EPCAM* gene; constitutional monoallelic methylation of the *MLH1* promoter	Mostly positive	Yes
**CMMR-D** (https://genturis.eu)	7.5	1 in 1 million new-borns	>90% of various of cancers at age 20	Constitutional biallelic germline mutation in DNA MMR genes	Positive	Yes
**Familial CRC X** (Choi et al., 2019; Garcia et al., 2022; Curtius et al., 2022; www.orpha.net)	Mid-50	1–2% of all CRC cases	Penetrance by age 70 years for the first CRC is 12.4% in men and 11.3% in women; lifetime CRC risk ~30%	MMR proficient with genetic etiology unknown; rare and potentially pathogenic alterations identified in putative candidate genes (*OGG1* and *FAN1*) and many other cancer-related genes	Negative	Yes
**Sporadic dMMR CRC** (Olave and Graham, 2021; Glasgow et al., 2022**)**	~65	12% of all CRC	No data found	Somatic hypermethylation of *MLH1* and commonly harbor *BRAF* mutations	Positive	No
**Lynch-like syndrome** (Pico et al., 2020; Martínez-Roca et al., 2022)	~55	Up to 50% of cases of suspected LS	Between LS and sporadic CRC	Loss of expression of MMR proteins by IHC in the absence of a germline MMR mutation; absence of *BRAF* mutation or *MLH1* hypermethylation; rare biallelic somatic mutations in the MMR genes	Positive	Mixed

## 3 Prevention

### 3.1 Prevalence of LS in general population

It is important to raise awareness of cancers associated with MMR deficiency. This is because ~1 in 650 individuals harboring a pathogenic or likely pathogenic variant in LS-associated MMR genes are found in the UK (46) with 100,000–150,000 individuals predicted with this condition; however, only 5% of patients with LS are aware of the diagnosis in the UK (47). In the United States, Canada, and Australia, it is estimated that 1 in 279 of the population carry mutations in MMR genes (37). The big discrepancy in prevalence between these two reports is speculated to be caused mainly by the selection of study cohorts, the former being 49, 738 participants with whole exome sequencing data available from UK Biobank; whilst the latter studied the families of 5,744 CRC cases from population cancer registries and apparently overestimated the prevalence. The other major difference between these studies is that the latter study did not filter out the benign or likely-benign gene variants and the variants with uncertain significance. In a very recent study, it is reported that LS affects about 1 in 354 of the US population (48). In this study, Park *et al*. reported that pathogenic variants in *MSH6* and *PMS2* genes account for the majority of the cases. Because families that meet stringent criteria for LS identification often have germline mutations in *MLH1* and *MSH2* [40% and 34%, respectively (49)], the inclusion of low penetrance pathogenic variants with atypical LS phenotype in the prevalence calculation as well as the use of an aged study cohort (median age being 58 years) could have introduced bias to the study. Albeit all three studies have their limitations, it is clear that LS carries high cancer risks and affects a large population worldwide.

### 3.2 Surveillance

#### 3.2.1 Colonoscopic surveillance

Colonoscopic screening and the removal of adenomas have been reported to reduce the risk of CRC development and decrease overall mortality by about 65% in LS families (50); therefore, preventative surveillance in asymptomatic patients using colonoscopy is recommended every 2 years in the UK, starting at the age of 25 years for *MLH1* or *MSH2* pathogenic gene carriers or 35 years for *MSH6* or *PMS2* carriers (47). Increasing surveillance frequency does not reduce the lifetime risk of CRC and there are three hypotheses to explain the unaltered rate of interval CRC (14): 1) CRC in LS develops through accelerated tumorigenesis pathways compared with sporadic CRC; 2) proximal and flat adenomas in LS are difficult to detect, let alone be removed on colonoscopy; 3) LS-associated CRC may have a unique, non-polypous carcinogenesis pathway. Advanced imaging techniques and high-quality colonoscopy surveillance with adequate bowel preparation are of utmost importance for CRC prevention in LS; however, currently colonoscopy is highly dependent on the skill of endoscopists and is subject to quality variability (51). From April 2023, a national quality-assured surveillance program has been delivered in England to improve performance and ensure high-quality colonoscopy (47). Real-time artificial intelligence (AI)-assisted techniques are promising approaches to potentially improve endoscopic surveillance in LS patients especially for the detection of flat polyps and adenomas (14, 52).

Colonoscopic screening every 3 years has been suggested for first-degree relatives of Lynch-like syndrome patients (53). Due to the wide spectrum of malignancies associated with CMMR-D, no clearly defined recommendations regarding methods and frequency are currently available for its surveillance (33). Current guidelines for average risk patients recommend starting at age 45 with repeat every 10 years if no adenoma is found (1).

#### 3.2.2 Other screening methods

Hereditary cancer susceptibility prediction models, i.e., PREMM5 and PREMMplus, have been developed by Dana-Farber Cancer Institute (https://blog.dana-farber.org) investigators to identify individuals who should be tested for LS based on their response to an online questionnaire. The probability that a person carries a mutation in any of the commonly seen LS-associated genes in their germline can be calculated using the information provided regarding biological sex, age, personal and family history of cancer. Individuals whose risk is ≥2.5% are recommended for genetic counseling and testing (54).

An ongoing trial, called the CORAL study (NCT05410977), is evaluating a non-invasive at-home DNA screening technique that uses blood and stool samples to detect CRC or advanced neoplasia in LS patients.

### 3.3 Preventative approaches

#### 3.3.1 Chemoprophylaxis

Daily aspirin administration for at least 2 years in patients below 70 years of age who are diagnosed with LS may reduce long-term CRC risk (www.nice.org.uk/guidance/ng151). Currently, it is not recommended in every country and caution should be taken in specific clinical circumstances in addition to the assessment for optimal prescription dose (14, 55).

#### 3.3.2 Vaccine

Vaccines that harness the immune system to prevent cancers in LS patients are being evaluated in a clinical trial (NCT05419011). This phase IIb trial tests whether the combination of trivalent adenovirus-5 (Tri-Ad5) vaccines and IL-15 superagonist nogapendekin alfa inbakicept (N-803) works to prevent colon and other cancers in participants with LS. As mentioned previously, LS-associated tumors have a high somatic mutation rate and hypermutations can occur in repetitive protein coding sequences resulting in the production of abundant frameshift peptides (FSP), which are novel antigens that can trigger strong immunogenic anti-tumor responses (56). A phase I/IIa clinical trial in humans with dMMR cancer showed that FSP neoantigen-based vaccines can consistently induce humoral and cellular immune responses and may be a promising approach for treatment and even prevention of dMMR cancer (57). Preclinical findings in a LS-associated mouse model also support a strategy of recurrent neoantigen vaccination for potential LS cancer prevention (58).

## 4 Diagnosis

Currently, diagnosis of LS is conducted using a combination of clinical phenotype, routine tumor pathology and genetic screening practices (9). Histologically, MMR-deficient tumors in LS patients tend to have a right-sided location with the presence of tumor infiltrating lymphocytes, Crohn's-like lymphocytic reaction, mucinous/signet-ring differentiation, or medullary growth pattern (9, 59). Since 2017, PCR detection for the presence of MSI and the loss of MMR protein expression on immunohistochemistry (IHC) are recommended by the National Institute of Health and Care Excellence (NICE) as a universal screening test for every case of CRC regardless of age and family history (40, 60). PCR and IHC testing for MMR pathway malfunction using biopsies only evaluate the phenotype of the tumor. Patients with defective MMR in the tumor specimen should be referred for genetic testing for potential LS diagnosis and counseling (www.nice.org.uk/guidance/dg27). Rectal cancer patients often undergo neoadjuvant therapy to shrink the tumor before resection, which may affect the IHC results. Next-generation tumor sequencing (NGS), as an alternative to sequential MSI or IHC testing followed by germline confirmation, may shorten the time of diagnosis and influence treatment decision-making (1).

Tumors with a loss of or an abnormal MLH1 expression routinely require a further test to detect *MLH1* promotor methylation (*MLH1*-PM) for the possible diagnosis of sporadic CRC. However, a rare case with concomitant presence of *MLH1*-PM and *MLH1* germline mutation reveals that the presence of *MLH1*-PM should not automatically rule out the diagnosis of LS. Additionally, constitutional methylation of *MLH1* as well as inactivation of the wild-type allele in LS tumors caused by *MLH1*-PM have both been reported (61). Furthermore, *MLH1*-PM and germline pathogenic variants in other MMR genes are not mutually exclusive (62). If a tumor shows abnormal IHC/MSI and is negative for *MLH1*-PM and *BRAF* mutation, the patient is eligible for genetic testing for LS. If the IHC result is abnormal for proteins other than MLH1, the patient may be immediately eligible for genetic testing. Any patient diagnosed with CRC under the age of 40 years may be referred for germline testing directly without MSI/IHC examination due to their high probability of having LS (47, 63). Asymptomatic patients among families with a confirmed pathogenic MMR variant can be referred for cascade genetic testing directly (14). Detailed diagnostic workflow charts can be found in a handbook published by NHS England (63).

CMMR-D may be diagnosed for patients with malignancies meeting one or more of the following criteria: presenting café-au-lait macules and/or other signs of NF1 and/or hypo-pigmented skin lesions; descendent from consanguineous parents; showing family history of LS-associated tumors and/or childhood cancer in siblings; or the existence of second malignancy (33). The diagnostic confirmation procedure is the same as it is for LS which involves MSI and IHC testing followed by gene-specific mutation analysis (33).

## 5 Treatment

Because sporadic CRC patients do not harbor germline MMR mutations for cancer predisposition, there should not be specific treatment concerns for these patients as with LS. Treatment for rare conditions associated with dMMR are not covered in this review due to the scarcity of published reports and guidance. Depending on the cancer stage at diagnosis, a treatment plan may include surgery, chemotherapy, radiotherapy, immunotherapy or a combination of two or more of these regimens (64).

### 5.1 Polypectomy and surgical resection of CRC

Currently, endoscopic polypectomy follows guidance for non-LS colorectal polyps, and it is critical to increase complete removal rates especially for sessile serrated polyps (14).

Surgical resection is the most common curative treatment for early-stage, localized colorectal lesions that involves the removal of the tumor and some of the surrounding tissues. Current surgical management for CRC is either a segmental or extended colectomy. For patients with high penetrance pathogenic variant(s) in *MLH1* or *MSH2*, extended colectomy may be considered to reduce risk of metachronous CRC, whereas for those with pathogenic variant(s) in *MSH6* or *PMS2*, standard segmental resection should be more beneficial (65). According to the results of a meta-analysis that compares the metachronous CRC risk in LS patients who underwent either segmental (*n* = 1,119) or extended colectomy (*n* = 270), segmental colectomy was associated with a significantly higher relative risk of metachronous CRC than the extended colectomy (22.4% vs. 4.7%) (66). Similar results were reported by another separate study (67). An extended colonic resection may reduce the need for further surgery, hospital stay and associated cost; however, no significant benefit association with mortality could be identified for this approach (66). For primary CRCs, UK guidelines consider these two modalities as equal, whereas European guidelines advocate segmental resection unless there are metachronous tumors (14). Many other factors need to be taken into consideration and the treatment decision should be “*a patient-centered, multidisciplinary approach, taking into account patient wishes, gene-specific risk, comorbidities and age”* (47). Because of the location and anatomical complexity, rectal cancers are treated differently from colon cancers. For early stages (stage 0 and I), segmental resection may be a better recommendation (1). Locally advanced (stage II and III) and metastatic (stage IV) rectal cancers are covered in 5.2, 5.3 and Table 2.

**Table 2:** Current treatment methods for dMMR colorectal tumors at different stages and some of those under investigation.

**Colon cancer** (Neoadjuvant immunotherapy, using anti-PD-1 and anti-CTLA-4, is being evaluated for stages I-III in the NICHE trials)	
**Early stages (0-I):** generally have better prognosis than MSS/pMMR tumors and may be removed by polypectomy or surgical resection	
**Locally advanced stages**	
Stage II	Surgery alone is normally sufficient. Generally do not benefit from 5-FU-based chemotherapy (e.g., FOLFOX). Chemotherapy may be avoided if no other high-risk features present. Oxaliplatin-based regimens can be used for high-risk cases.
Stage III	Complete removal of the tumor with regional lymph node dissection. Standard adjuvant chemotherapy still in use (FOLFOX or CAPOX) to reduce recurrence risk similar to MSS cases. 5-FU alone is not sufficient. Adjuvant chemotherapies may have long-term negative outcome, investigations with or without immunotherapy are on-going to evaluate their efficacy.
**Metastatic stage (Stage IV)**	
Immune Checkpoint Inhibitors (ICIs) are now first-line treatment, such as pembrolizumab monotherapy or nivolumab plus ipilimumab dual checkpoint blockade for previously treated (second-line or beyond) or more aggressive tumors. Some patients respond well to immunotherapy with longer PFS; however, some are resistant. Chemotherapy may be considered if ICIs fail. Radiotherapy is rarely used except for palliative care.	
**Rectal cancer**	
**Early stages (0-I):** local excision	
**Locally advanced stages (stage II and III)**	
Typically involves neoadjuvant chemoradiotherapy and surgery for stage II. If chemotherapy is needed for stage III, use oxaliplatin-based regimens in the adjuvant setting. Under study, but some cases benefited from neoadjuvant anti-PD-1 (e.g., dostarlimab) monotherapy that can be used as first-line treatment.	
**Metastatic stage (stage IV)**	
Same as colon cancers, some patients benefit from checkpoint inhibitors; however, immune-related adverse events require early recognition and management. Chemotherapy is used if immunotherapy fails but it is no longer the preferred first-line for dMMR patients.	

### 5.2 Chemotherapy

For CRC treatment with no consideration given to MSI/MMR status, commonly prescribed chemotherapy agents include pyrimidine analog 5-fluorouracil (5-FU) and oxaliplatin that act to limit tumor cell division (68). They are commonly used along with leucovorin in a combination known as FOLFOX (www.cancer.gov). These drugs have a high response rate in CRC; however, many patients are resistant possibly due to the existence of alternative pathways currently unknown (1).

Fluorouracil (FU)-based adjuvant therapy has been shown to significantly improve disease-free survival (DFS) for patients with MMR proficient (pMMR) tumors. However, patients with dMMR tumors receiving 5-FU showed no improvement in terms of DFS when compared with those randomly assigned to surgery alone. Moreover, in patients with stage II dMMR tumors, this adjuvant therapy was associated with significantly reduced overall survival (69). Therefore, patients with stage II dMMR colon cancers may not benefit from single-agent, fluoropyrimidine-based adjuvant therapy. Nevertheless, patients with stage III tumors showed a significant survival benefit from fluoropyrimidine-based adjuvant treatment regardless of MSI status (30).

Adding oxaliplatin to FU-based therapy improves disease-free and overall survival in patients with stage III disease, whereas no overall survival benefit in unselected patients with stage II disease was observed from adding oxaliplatin (69). Similarly, no benefit was shown in oxaliplatin-based adjuvant chemotherapy in stage II dMMR tumors when compared to surgery alone in either high-risk or low-risk CRCs (70). More recently, data from several retrospective analyses showed that the benefit of oxaliplatin-based adjuvant chemotherapy (e.g., capecitabine plus oxaliplatin-CAPOX) appears to be independent of MSI status and is favored for stage III tumor regardless of MSI status (30).

For advanced or metastatic CRC with MSI-H/dMMR, immunotherapy is now the recommended first-line treatment (Section 5.4); however, chemotherapeutic agents are still in use for high-risk or late stage MSI-H/dMMR CRC in both sporadic and LS cases (14). Therefore, CRC patients who are considered for chemotherapy should have the tumor MMR status assessed. The effectiveness of chemotherapy for LS patients at different cancer stages may need to be comprehensively reviewed and clinical guidelines be updated accordingly.

### 5.3 Radiotherapy

#### 5.3.1 Adverse effects of radiation

Radiotherapy is generally provided during cancer treatment to either kill or control the growth of malignant cells. It may be used alone for localized tumor or synergistically with surgery, chemotherapy, or immunotherapy for CRC depending on the confinement and progression of the tumor (71). Nevertheless, ionizing radiation (IR), such as X-rays and gamma rays used for radiotherapy, can cause damage to DNA directly through electron ejection that disrupts the molecular structure of DNA or indirectly by the production of reactive oxygen species through water radiolysis (72). It has been widely reported that the detrimental effects of IR are not restricted in the targeted cells, various IR-induced biological effects may occur in non-irradiated bystander or even distant cells (73). Thus, it is essential to ensure that the benefits of radiation therapy significantly outweigh the harm in clinical practice.

Radiation initiates a complex network of DNA damage response pathways and MMR proteins play important roles in DNA damage repair including IR-induced damage (11). Despite a paucity of evidence, radiotherapy may cause mutation or alteration in the regulation of MMR genes leading to further tumorigenesis. LS is recessive at the cellular level with a heterozygous mutated allele in the MMR gene and secondary damage to the wild-type allele in separate events during radiotherapy may lead to the loss of function and subsequent development of a somatic tumor (12). Currently, no data or longitudinal observational studies for the impact of radiation exposure on humans with LS can be identified. However, mouse model studies have demonstrated the association between dMMR and accelerated tumor development (74) as well as increased CRC risk and tumor number (75) in animals exposed to radiation.

#### 5.3.2 Radiation and MMR

The mechanisms through which IR influences MMR gene and protein expression are unknown, but they may include genome-wide epigenetic modifications and microRNA-mediated pathways (12). Nevertheless, it is clear that the expression of MMR genes is highly dependent on the specific gene, age and gender of the patient, radiation dose and dose rate, type of exposure (i.e., acute or protracted), duration between exposure and investigation among many other unidentified factors (76, 77). It has been shown by reverse transcription quantitative real-time PCR that therapeutic doses of X-rays can affect the expression of MMR genes in human colorectal cancer cell lines associated with LS (i.e., *MLH1*-deficent: HCT116, SW48; *MSH2*-deficient: LoVo; and MMR-proficient: HT29). In reference to a human house-keeping gene, hypoxanthine guanine phosphoribosyltransferase 1 (HPRT1), *MLH1* and *MSH6* genes were stably expressed even after a daily exposure of 2 Gy for five consecutive days to mimic fractionated radiotherapy in all associated cell lines. In contrast, significantly up- or down-regulated expression was observed for the *MSH2* gene in HCT116 and SW48, respectively. Similarly, *PMS2* is another gene that may be subject to radiation-induced change in expression. For protein expression investigated using Western Blotting, MLH1 was found to be stable, whilst MSH2 was significantly affected by radiation dose (78), which is in agreement with another study using nasopharyngeal carcinoma CNE-1 cells (79). Due to the inherent limitations associated with cancer cell lines especially genomic instability, clinical samples from LS patients may provide the cell- or organoid- based materials for further studies to overcome these limitations and to enable the investigation of radiation effects in LS patients.

#### 5.3.3 Neoadjuvant radiotherapy

For locally advanced rectal cancers, current treatment modality includes: 1) neoadjuvant long-course radiotherapy (RT) combined with radiosensitizing chemotherapy; 2) neoadjuvant short-course RT alone followed by adjuvant chemotherapy and 3) total neoadjuvant therapy with induction chemotherapy followed by chemoradiotherapy (80).” Neoadjuvant radiotherapy has been shown effective in downsizing or downstaging large tumors and the use of neoadjuvant chemoradiotherapy (CRT) improves local control and has lower toxicity than postoperative CRT (81). NCCN (National Comprehensive Cancer Network) Guidelines recommend neoadjuvant approaches for Stage II–III (including node-positive T2) rectal cancers (82). However, complications, side effects, and toxicity have been reported (68) in addition to a wide variation in the extent of radiation induced tumor regression (83).

### 5.4 Immunotherapy

dMMR tumors exhibit higher levels of immunogenicity than pMMR tumors because MMR deficiency allows accumulation of mutations in microsatellite sequences leading to translational frameshifts and the generation of FSPs (14). FSPs can serve as “neoantigens” to stimulate the anti-tumor host immune response and the immunoreactive nature of MSI-H/dMMR CRCs has prompted the use of immunotherapies that have been proven exceptionally effective in some of the LS patients. A significant recent advance in the treatment of CRC carcinomas that exhibit specific genetic markers, such as MSI-H or dMMR, is the immunotherapy using immune checkpoint inhibitors (ICI) that offers long-lasting responses and potential significant survival benefits (www.nice.org.uk/guidance/ta709). The neoadjuvant use of ICIs is paving the way for non-surgical interventions potentially transforming the management of CRC in LS patients (55).

One group of efficacious ICIs act to inhibit the binding of programmed death-ligand 1 (PD-L1) on tumor cells with its receptor, programmed cell death protein 1 (PD-1), on T-cells. The interaction of these cell surface proteins can reduce T-cell function signaling and prevent the immune system from attacking the tumor cells (84). Checkpoint antibody inhibitors, such as monoclonal anti-PD-1/PD-L1 antibodies function as tumor suppressing factors through modulation of interaction between immune cells and tumor cells (85). In a study that evaluates the clinical activity of pembrolizumab, an anti-PD-1 ICI, it was found that for patients with dMMR CRC, the immune-related objective response rate (ORR) and immune-related progression-free survival (PFS) rate were significantly higher than those for CRC with pMMR and MSS (86). Pembrolizumab has also been reported to significantly extend PFS of metastatic CRC patients with dMMR in comparison to chemotherapy with fewer treatment-related issues (87). Pembrolizumab is now NICE (www.nice.org.uk/guidance/ta709) and US Food and Drug Administration (FDA; www.fda.gov) approved for first-line treatment of advanced or unresectable CRC with dMMR/MSI-H. Another PD-1 inhibitor, nivolumab, is also NICE- and FDA-approved and can be used in combination with ipilimumab for previously treated or more aggressive metastatic CRC (www.nice.org.uk/guidance/ta716) (34). Ipilimumab is one of the cytotoxic T-cell lymphocyte-4 (CTLA-4) inhibitors and this dual checkpoint blockade for first-line treatment is more effective however with increased toxicity (88–90). In addition, pembrolizumab and nivolumab have been approved by FDA as the second-line treatment for metastatic dMMR/MSI-H CRC (91). More ICIs have been approved with time or are being evaluated; however, the benefit of ICIs is currently limited to metastatic disease, ongoing studies evaluating ICIs as neoadjuvant or adjuvant therapy in earlier-stage dMMR CRC may further improve the treatment of LS patients (88).

For sporadic MSI tumors developed due to *MLH1*-PM, ICI-based immunotherapy may result in different treatment responses as reported in one of the pioneering studies that LS cancer patients had an ORR of 27% compared to an ORR of 100% for sporadic MSI tumors (86). However, systematically collected data indicated no significant difference in terms of ORR and PFS between LS and sporadic MSI/dMMR tumors treated with FDA- or European Medicines Agency-approved immune checkpoint-based drugs targeting either PD-1, PD-L1, or CTLA-4 at various treatment lines (34). Similar findings were presented by Eslinger et al. (92) that MSI-H CRC patients treated with ICIs have similar outcomes for germline and somatic MMR mutations. Due to limited sample sizes and large confidence intervals, further investigation is still required (34).

Immune checkpoint blockade (ICB) treatment for first cancers may decrease the incidence of second primary cancers (93). It has recently been reported that early stage dMMR cancers were more responsive to ICB than at metastatic stage. However, it was also revealed in this study that ICB treatment for LS-associated tumors did not eliminate the development of new neoplasia especially those in skin. Nevertheless, this treatment was found to be associated with significantly reduced development of serious intestinal tumors in contrast to the control group treated with chemotherapy (15).

It should be noted that immune checkpoint inhibitors can potentially cause the immune system to attack normal organs and tissues. Many immune-related adverse events cause little discomfort to patients if detected early. However, they can affect any organ and be potentially life-threatening. Serious side effects typically occur in < 5% of patients, but certain mild side effects can occur in up to 30–50% of them. Adverse side effects are more likely to occur if the patients take more than one kind of immunotherapy or immunotherapy combined with other types of anti-cancer treatments (https://www.nccn.org). More than 50% of patients experience moderate to severe side effects with ipilimumab and nivolumab. Anti-CTLA4 drugs usually have more adverse effects (up to 80%) compared to anti-PD1 (27%) and anti-PDL1 (17%) drugs (https://www.cancerresearchuk.org). Additionally, side effects may appear shortly after the beginning or within the first couple of months of the treatment or even after the completion (https://www.nccn.org), and it is currently not possible to predict which patients develop immune-related adverse events. Some patients with dMMR CRC may have intrinsic or acquired resistance to immunotherapy due to molecular mechanisms that are yet to be fully elucidated (88, 91). Ongoing clinical trials investigating combinations of ICIs with chemotherapy and/or biological treatments may increase response rates in resistant dMMR/MSI-H CRC (91). MSS phenotype has been found in 36% of LS tumors resulted from *MSH6* and *PMS2* alterations (34). MSS tumors are normally non-colorectal and may not respond to immunotherapy due to the lack of neoantigen presentation.

Table 2 summarizes the currently used treatment modalities and those under investigation for dMMR colon and rectal cancers at different stages. Because of tumor heterogeneity and the lack of solid molecular markers to select responsive cases, a “watch-and-wait” strategy may be beneficial for all CRC treatment approaches and the combination of them (www.cancerresearchuk.org).

### 5.5 Other potential treatment methods

Emerging treatments, such as chimeric antigen receptor T cell therapy, T cell receptor alterations, the use of probiotics, cytokine therapy, and RNA-based therapies have been reported for CRC treatment. In addition, oncolytic viral therapies and natural products also showed promising results in the treatment of CRC (71). Whether any of these methods can be used for the treatment of dMMR CRC is dependent on further understanding of the pathophysiological and molecular mechanisms of LS.

## 6 Service and management

For the care and management of CRC patients, it is critical to establish and adhere to national guidelines to avoid quality variability (94). A multidisciplinary team is required for the life-time support and management of LS patients and their families with expertise covering gastroenterology, surgery, oncology, specialist nursing care and others appropriately affiliated to regional genetics services (47).

MMR pathogenic variants are associated with great variability in penetrance and expressivity (14); therefore, the lifetime risk of CRC development is also gene-specific depending on the underlying MMR pathogenic variant. It is ~44%−70% in patients with *MLH1* variant; 42%−46% and 18%−20% in *MSH2* and *MSH6* variant carriers, respectively; and around 10%−13% for *PMS2* variant carriers. Consequently, clinical management should be arranged according to the specific need of different pathogenic variant carriers (47). The establishment of the Prospective Lynch Syndrome Database, together with the InSiGHT database (www.insight-group.org), has facilitated the prospective study of cancer incidence, overall survival and the effects of interventions in carriers of MMR pathogenic variants categorized by age, gene and gender, which may enable evidence-based personal healthcare (95). NGS assisted diagnosis in clinical practice will improve the detection rates of LS and therefore allow for more effective and personalized management (14).

Similar to other CRC patients, enrolment in screening via registration may not only reduce CRC mortality (96), but it may also allow streamlined organization of carriers in families as well as information dissemination for LS patients. Furthermore, it facilitates continuity of care, access to genetic counseling, testing and involvement in clinic trials for the patients and enables epidemiological and molecular genetic studies, blood and/or tissue collection for biobank and the research for preventive and therapeutic techniques (47).

## 7 Conclusion

Patients with MMR deficiency require lifelong, coordinated multidisciplinary care; nevertheless, there are no clear streamlined and standardized strategies for the treatment and management of these patients at present. A personalized approach for individuals with gene-specific LS may be possible with the availability of NGS analysis in the future that can be used for the management of cancers as well as long-term cancer risks. Continued and targeted research is essential to optimize cancer prevention and treatment and to improve the quality of life for people with LS. Overall, it is important to raise awareness for this condition that is associated with high prevalence and high cancer risk. In combination with early diagnosis and intervention, it is possible to reduce cancer burden and mortality in these patients. This review provides the most updated information for the prevention, diagnosis, and treatment of CRC with MMR deficiency. It can be used by LS patients and asymptomatic pathogenic MMR mutation carriers as well as their families for educational purpose. In addition, it can potentially inform researchers and assist medical staff with clinical decision making.

## Author's note

The Health Protection Research Unit (HPRU) in Radiation Threats and Hazards is part of the National Institute for Health and Care Research (NIHR) and a partnership between UK Health Security Agency and Imperial College London, in collaboration with the King's College London, Institute of Cancer Research and MRC Toxicology Unit at University of Cambridge. We are a multidisciplinary team undertaking the highest quality research on the health effects of exposures to hazardous radiation to improve assessment, management and control of risk to humans. Our aim is to support UKHSA in delivering its objectives and functions. Follow us on Twitter: @HPRU_RTH.
